# In-Line Monitoring the Degradation of Polypropylene under Multiple Extrusions Based on Raman Spectroscopy

**DOI:** 10.3390/polym11101698

**Published:** 2019-10-16

**Authors:** Xuemei Guo, Zenan Lin, Yingjun Wang, Zhangping He, Mengmeng Wang, Gang Jin

**Affiliations:** 1 National Engineering Research Center of Novel Equipment for Polymer Processing, South China University of Technology, Guangzhou 510641, China; guoxm2710@163.com (X.G.); lzn557968@163.com (Z.L.); wangyj84@scut.edu.cn (Y.W.); mezhphe@mail.scut.edu.cn (Z.H.); memmwang@scut.edu.cn (M.W.); 2 Key Laboratory of Polymer Processing Engineering of Ministry of Education, South China University of Technology, Guangzhou 510641, China; 3 Guangdong Provincial Key Laboratory of Technique and Equipment for Macromolecular Advanced Manufacturing, South China University of Technology, Guangzhou 510641, China

**Keywords:** in-line monitoring, Raman spectroscopy, degradation, multiple extrusions, quantitative

## Abstract

Polymer degradation is a common problem in the extrusion process. In this work, Raman spectroscopy, a robust, rapid, and non-destructive tool for in-line monitoring, was utilized to in-line monitor the degradation of polypropylene (PP) under multiple extrusions. Raw spectra were pretreated by chemometrics methods to extract variations of spectra and eliminate noise. The variation of Raman intensity with the increasing number of extrusions was caused by the scission of PP chains and oxidative degradation, and the variation trend of Raman intensity indicated that long chains were more likely to be damaged by the extrusion. For the quantitative analysis of degradation, the partial least square was used to build a model to predict the degree of PP degradation measured by gel permeation chromatography (GPC). For the calibration set, the coefficient of determination (*R*^2^) and the root mean square error of cross-validation (RMSECV) were 0.9859 and 1.2676%, and for the prediction set, *R*^2^ and the root mean square error of prediction (RMSEP) were 0.9752 and 1.7228%, which demonstrated the accuracy of the proposed model. The in-line Raman spectroscopy combined with the chemometrics methods was proved to be an accurate and highly effective tool, which can monitor the degradation of polymer in real time.

## 1. Introduction

The extrusion process is a highly efficient, continuous, and low-cost process that is widely used in polymer processing and is one of the most important forms of plastic material processing. During the extrusion process, polymer degradation is a common problem. The unavoidable thermo-mechanical effect induces a change in the properties, which have a strong influence on the polymer lifetime. The polymer is influenced by mechanical stress, oxygen and heat, and, therefore, polymer degradation during extrusion is caused by a combination of mechanical, chemical, and thermal degradation, and is quite a complex process [1].

In the case of polypropylene (PP), the scission of the molecular chain is primarily induced by the mechanical stress, including shear and tensile stresses during the extrusion process, and the dramatic variation of the PP backbone will lead to the decrease of molecular weight [2]. Moreover, during the extrusion process, chemical degradation is mainly induced by oxygen, which usually results in the oxidative reaction of the polymer. Free radicals, initiated in the initiation, react with other PP backbones, and in the termination stage, they are oxidized as ketone or ester functional products [3]. Thus, based on the oxidative degradation mechanism of PP, the degradation of PP is terminated with products of oxygen-containing functional groups. These products, split from the PP backbone, also lead to the decrease in molecular weight. In addition, the inherent stabilization of the polymer is destroyed by the heat provided from the extrusion [4], and the oxidation reaction occurring in the extrusion is accelerated by the elevated temperature. Therefore, during the extrusion process, thermal degradation is one of the important parts in polymer degradation when the polymer undergoes elevations in temperature.

To protect the mechanical properties of the product, the number of extrusions must be limited to preserve the desired properties of the polymer. The degradation of the polymer under multiple extrusions has been evaluated by traditional measurements, such as the mechanical properties test [5,6], gel permeation chromatography (GPC) [7,8], and rheological measurement [9,10,11]. In order to characterize the degradation mechanism of the polymer, a combination of several off-line measurement methods is often required. However, these measurements are all off-line methods, which are destructive, hysteretic, time- and cost-consuming. In recent work, in-line measurements for monitoring polymer degradation during the extrusion have been widely researched. Wang et al. [12] use ultra-violet/visible (UV-vis) spectroscopy to in-line monitor the degradation of poly (L-lactic acid) (PLLA) in the twin screw extruder, and they find a good correlation between the degradation rate and the UV-vis absorption of PLLA. Montano-Herrera et al. [13] predict the degree of polyhydroxyalkanoate (PHA) degradation in the laboratory-scale extruder using the Near-Infrared (NIR) spectroscopy. Hamester et al. [14] develop an in-line colorimeter, which can in-line quantify and measure the color change in PP due to multiple extrusion degradations. In-line measurements can characterize the molten polymer in real time and, ultimately, guide the adjustment of processing parameters to preserve the desired properties of products. 

Raman spectroscopy, as a robust, rapid, and non-destructive analytical technique, is a reliable tool for in-line monitoring. During the hot-melt extrusion, the in-line Raman spectroscopy can monitor melt characteristics, such as component content [15,16], molecular interactions [17,18], and phase state [19,20]. However, the above studies have been published in the field of pharmaceuticals, the in-line Raman spectroscopy has not been widely used in the field of polymers. Raman spectroscopy, the measurement of the Raman scattering intensity and wavelength, provides abundant information about molecular vibrations [21]. The intensity of Raman scattering is proportionate to the number of molecules excited by stimulated light, which is the basis for quantitative analysis of Raman spectroscopy [22]. Therefore, the in-line Raman spectroscopy can monitor the degradation of the polymer during the extrusion. In combination with the chemometrics methods, the degree of the polymer degradation can be also measured by the in-line Raman spectroscopy to monitor the quality of PP in real time.

The purpose of this work is to utilize the in-line Raman spectroscopy with the chemometrics methods to monitor the degradation of PP under multiple extrusions in real time. For this purpose, at first, we set up a system of in-line Raman spectroscopy to monitor the melt during the extrusion. To ensure that Raman spectra can be collected in a high-temperature and -pressure environment, the temperature–pressure-resistant shell was designed to protect the Raman probe. Then, raw spectra were pretreated by the chemometrics methods to extract variations of spectra, and off-line measurements were utilized to prove the correctness of these variations. At last, combined with the PLS model, in-line Raman spectra were utilized to predict the degree of PP degradation, as measured by gel permeation chromatography.

## 2. Experimental

### 2.1. Materials and Sample Preparation

The raw material PP (HD822CF) was supplied by Borealis, with a melt-flow rate (MFR) of 7.1632 g/10 min (230 °C, 2.16 kg) and the weight average molecular weight (*M*w) of 383,352. 

The PP was repeatedly extruded 15 times in the triple-screw extruder (IEGTC-25/40, POTOP, China). In parallel, the extrusion processing temperature was set at 190 °C and the screw speed was set at 200 rpm. After each extruding process, 16 granular samples were collected (including raw materials PP), in preparation for measurements to be carried out.

### 2.2. In-Line Raman Spectroscopy Measurement

In-line Raman spectra of 16 melting samples were collected by the in-line monitoring system of Raman spectroscopy, including a Raman spectrometer (QE65 Pro, Ocean Optics Inc., FL., Largo, USA), a 785 nm single longitudinal mode laser (Laser785-5HSB, BIAOQI, Guangzhou, China) and a Raman fiber-optic probe (RamanProbe II, InPhotonics, MA., Norwood, USA). 

**Figure 1:**
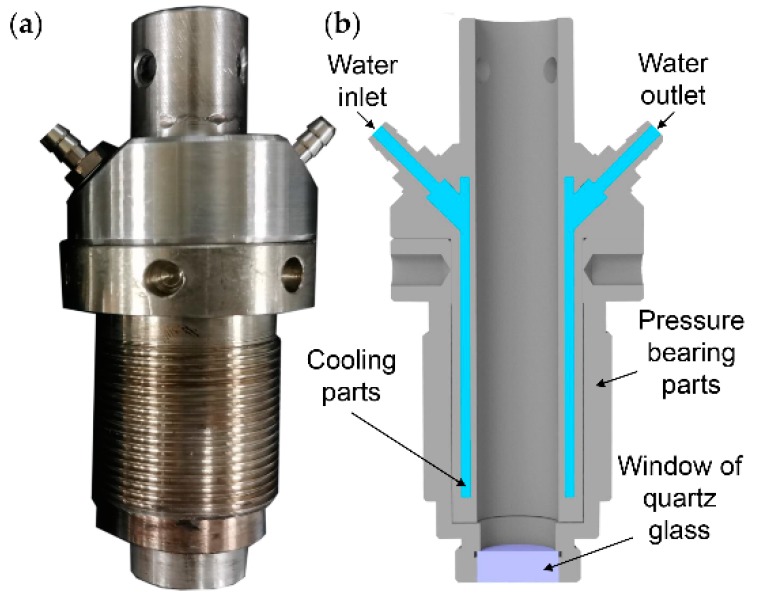
The high-temperature/pressure-resistant shell. (**a**) The physical photo, (**b**) the schematic diagram.

The granular sample was melted and stably delivered to the slit sample cell, which was installed on the front of the extruder. Because of the high-temperature and -pressure environment inside an extruder, a high-temperature/pressure-resistant shell (Figure 1) equipped with a water channel was designed to protect the Raman probe. The high-temperature/pressure-resistant shell is composed of pressure-bearing parts (external) and cooling parts (internal). A quartz glass window is adhered to the stainless-steel parts at the front with epoxy resin. The high-temperature/pressure-resistant shell not only cooled the Raman probe to ensure its regular operation, but also provided a window for the acquisition of the Raman signal in the extruder. The laser from the 785 nm laser was irradiated onto the molten sample through the window of the high-temperature/pressure-resistant shell along the excitation fiber of the Raman probe. The Raman signal generated from the sample passed through the window into the collection fiber of the Raman probe, and was, subsequently, collected by the Raman spectrometer. The Raman spectrum of each sample was averaged over 20 scans with a resolution of 4 cm^−1^ over the wavenumber range from 1600 cm^−1^ to 600 cm^−1^. In addition, the power of the laser was at a rate of 400 mW. To obtain a high signal-noise ratio (SNR), the distance of the laser spot to the melt surface was set 0 mm, and the single scan time was set to 3 s [23]. The raw spectrum was pretreated by the chemometrics methods to correct baselines and eliminate unwanted noise.

### 2.3. Off-Line Methods and Measurement

Gel permeation chromatography (GPC) was carried out by PL-GPC220 (Agilent Technologies, CA., Santa Clara, USA) at 150 °C with a sample concentration of 0.10 mg/mL. The columns used were three PLgel 10 mm Mixed-B type columns and set length of 950 mm. The RI detector was used. The eluent was trichlorobenzene (TCB). The injection volume was 200 μL and the flow rate was 1.0 mL/min. Calibration was made by narrow standard. The Mark-Houwink coefficients, K and alpha, of the samples were 14.1 × 10^−5^ mL/g and 0.70, respectively.

According to ASTM D1238, MFR was measured by the Melt Indexer (MP993, Tinius Olsen, PA., Horsham, USA) at 230 °C, 2.16 kg with the die diameter of 2.095 mm and the tube length of 8 mm.

The Fourier-Transform Infrared (FTIR) spectrum was observed from the 50 μm thin film of the sample by the Fourier-Transform spectrometer (Nexus 670, Thermo Nicolet Corporation, WI, Madison, USA). Infrared spectrum of the sample was recorded over the wavenumber range from 4000 cm^−1^ to 400 cm^−1^ at a resolution of 4 cm^−1^.

The mechanical tensile test was carried out to acquire the tensile property with dumbbell-shaped tensile specimens (5 specimens of each sample), using an Electronic Universal Material Testing Machine (5566, Instron, MA, Canton, USA), in accordance with ASTM D638, at a speed of 50 mm/min.

## 3. Results and Discussion

### 3.1. Analysis of In-Line Raman Spectra

**Figure 2:**
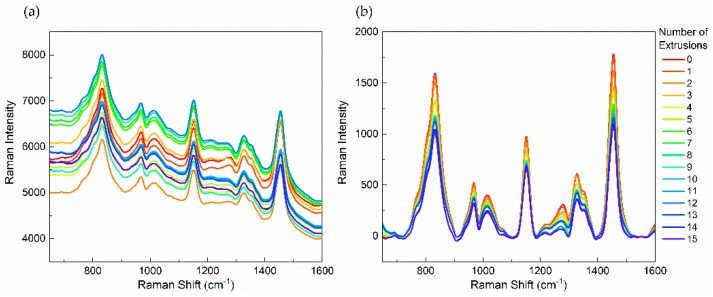
In-line Raman spectra of polypropylene (PP) with different numbers of extrusions. (**a**) raw spectra, (**b**) spectra pretreated by baseline correction.

The fluorescent background and the baseline drift are ubiquitous phenomena in the Raman spectroscopy measurement and can adversely affect the feature extraction of Raman spectral signals. Furthermore, it is difficult to distinguish the difference between Raman spectral intensities of various spectra only from the raw data. Therefore, an excitation wavelength of 785 nm is chosen, in which case, the excitation energy is generally insufficient to excite strong fluorescence [24]. It is also necessary to conduct baseline correction pretreatment. Baseline correction is performed through subtraction of a linear or polynomial fit of the baseline from the raw spectrum to remove tilted baseline variation caused by various noises. Before the analysis of Raman spectra, the automatic baseline correction (OMNICTM, version 8, Thermo Scientific, MA, Waltham, USA) of chemometrics methods is chosen to pretreat the raw spectrum to ensure the accuracy of spectral characteristic bands (Figure 2). 

**Figure 3:**
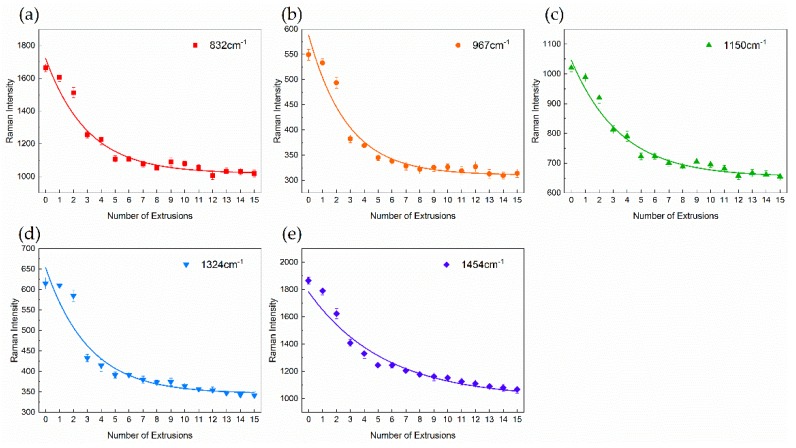
Variations and fitting curves of Raman intensity in PP main characteristic bands with different numbers of extrusions. (**a**) 832 cm^−1^, (**b**) 967 cm^−1^, (**c**) 1150 cm^−1^, (**d**) 1324 cm^−1^, (**e**) 1454 cm^−1^.

**Figure 4:**
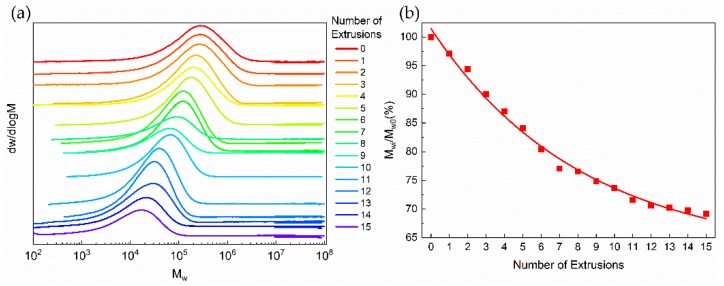
Results of GPC in PP with different numbers of extrusions. (**a**) Variation of Molecular weight distribution curve, (**b**) variation of *M*_wi_/*M*_w0_. M_Wi_ is the weight average molecular weight of the i-th extrusion.

**Figure 5:**
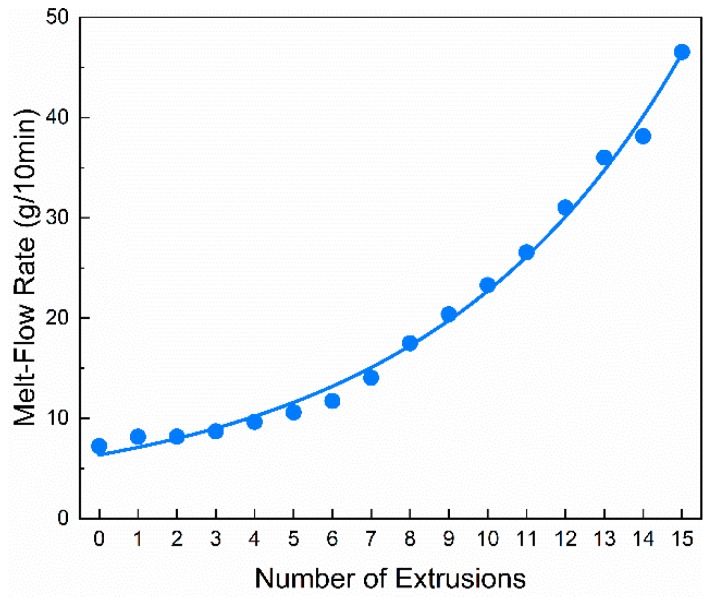
Variations of melt-flow rate (MFR) in PP with different numbers of extrusions.

**Figure 6:**
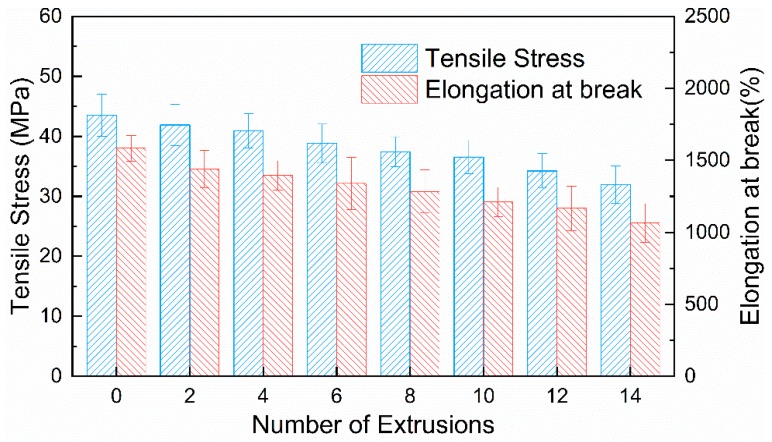
Variations of tensile properties in PP with different numbers of extrusions.

Vibrational modes for Raman characteristic Bands of PP are shown in Table 1. Variations of Raman intensity in PP main characteristic bands with different numbers of extrusions are shown in Figure 3. Raman intensities of 1324 and 1454 cm^−1^ bands gradually decrease with an increasing number of extrusions, indicating the decrease in the –CH_2_– group. In addition, the similar variation in spectral intensity has been observed in bands of 832, 967 and 1150 cm^−1^, suggesting the decrease of the C–C. These spectral variations show that chains of PP are damaged by the mechanical stress during the extrusion process. By means of multiple extrusions, the scission of the PP chain mainly results in the decrease in molecular weight [25,26]. As shown in Figure 4, it can be found that *M*_w_ decreases with the increasing number of extrusions, which indicates the scission of the PP chain. The molecular weight decreases as the degree of degradation increases, the number of chain scissions increases as the number of extrusions increase. Macroscopically, the scission of the molecular chain is reflected in the increase of the MFR [27] and the decrease of the tensile properties [5] as well. As shown in Figure 5 and Figure 6, with the increasing number of extrusions, the MFR increases and the tensile properties decreases, suggesting the scission of the molecular chain.

**Figure 7:**
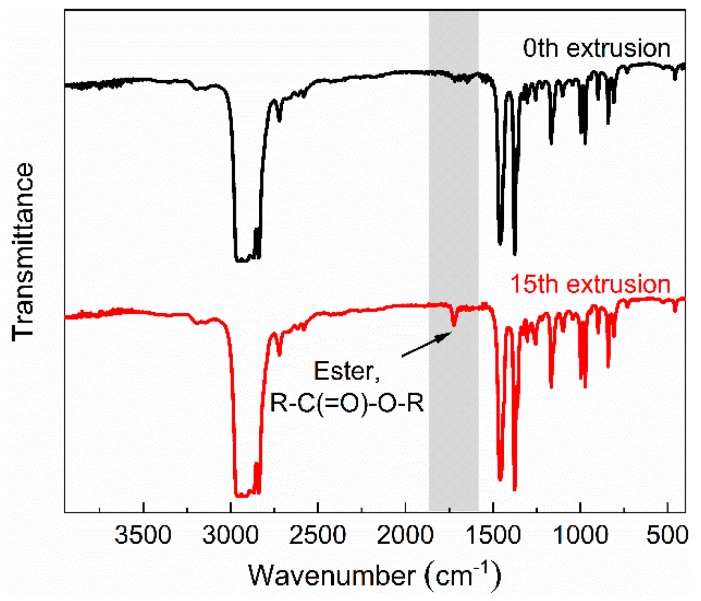
FTIR spectra of PP with different numbers of extrusions.

A similar variation in spectral intensity is observed in the band of –CH_3_ vibration. As shown in Figure 3, Raman intensities of 832, 967, 1324 and 1454 cm^−1^ bands show a clear decrease, suggesting the decrease of the –CH_3_ group. Because of the low activation energy of the alkyl radical of PP (80–110 kJ/mol), free radicals are easily initiated in an aerobic environment [29]. Considering that the extrusion process of PP is performed in a natural atmosphere, the decrease of the –CH_3_ group indicates that free radicals may be terminated with oxygen-containing functional groups instead of –CH_3_ groups. Therefore, oxidative degradation may happen in the extrusion. According to Figure 7, FTIR spectra of degraded PP for different numbers of extrusions, the ester functional group has a clear peak (1740 cm^−1^) in the sample of the fifteenth extrusion [3]. This result indicates that the PP has undergone oxidative degradation and esterification of carboxylic acids with alcohol has occurred at the termination stage.

Furthermore, as shown in Figure 3, the Raman intensity in all bands sharply decrease at first, and the difference of the Raman intensity decreases with the increasing number of extrusions. Decreases of the –C–C– and –CH_2_– group demonstrate the scission of the PP chain, thus, a similar variation trend is observed in the scission of the PP chain. This variation indicates that the rate of degradation gradually decreases with the increasing number of extrusions. Considering that long chains are more likely to be damaged by mechanical stress [30], the decrease of the degradation rate is explained as the scission of long molecular chains with an increasing number of extrusions [31]. According to Figure 4a, with an increasing number of extrusions, the molecular weight distribution curve shifts toward a low molecular weight, which shows a decrease in long molecular chains and an increase in short molecular chains. The result demonstrates that long molecular chains are more likely to be changed by the mechanical stress during the extrusion process. 

An in-line Raman spectroscopy measurement can show the variation of PP degradation in the extrusion, and the correctness of these variations is proved by various off-line measurements. This proves that an in-line Raman spectroscopy measurement can monitor the degradation in the extrusion in real time.

### 3.2. Degradation Assessment of PP

To assess the degradation of PP, it is necessary to specifically quantify the degree of degradation. Since the degradation in the extrusion mainly results in the decrease of the molecular weight, the degree of degradation was defined as the degree of molecular weight degradation, which was measured by the GPC, according to Equation (1).

(1) $$\mathrm{The}\;\mathrm{degree}\;\mathrm{of}\;\mathrm{degradation}\;=\;\frac{M_{wi}}{M_{w0}}\;\times 100\%$$

In Equation (1), *M*_Wi_ is the weight average molecular weight of the i-th extrusion. To eliminate undesired information and noise, using chemometric methods, the automatic baseline correction is chosen to pretreat the raw spectra before building the model. The intensity of Raman scattering is proportionate to the number of molecules excited by stimulated light. As shown in Table 2, linear correlation coefficients between Raman intensity of PP main characteristic bands and the degree of degradation are greater than 0.9, which shows a highly linear positive correlation between the Raman intensity of the PP main characteristic band and the degree of degradation. Thus, it is feasible that the degree of degradation is measured by in-line Raman spectroscopy. Since Raman main characteristic bands of PP are mainly concentrated in the 1600–600 cm^−1^, the range of 1600–600 cm^−1^ is chosen to develop the model. Sixteen samples are kept separate for model building and prediction testing. The 1st, 4th, 7th, 10th and 13th samples function as a blind dataset for testing the model accuracy, and the rest of the samples are used to build the model. 

To obtain an accurate prediction of the degree of degradation, the partial least squares (PLS) is utilized to develop a linear calibration model. PLS, a combination of multiple linear regression, canonical correlation analysis and principal component analysis, is widely used in spectral multivariate calibration analysis. PLS modeling includes matrix decomposition and regression. The matrix decomposition is shown in Equations (2) and (3).

(2) $$X=TP^{T}\;+\;E_{X}\;=\;\sum_{k=1}^{f}t_{k}p_{k}^{T}\;+\;E_{x}$$

(3) $$Y=UQ^{T}+E_{Y}=\sum_{k=1}^{f}u_{k}q_{k}^{T}+E_{Y}$$

In Equations (2) and (3), X and Y are the Raman spectral matrix and the degree of degradation matrix, respectively; T and U are the score matrices of X and Y; P and Q are the load matrices of X and Y; E_X_ and E_Y_ are PLS fitting residual matrices of X and Y; f is the number of the main factor. Then, according to Equations (4) and (5), the T and U matrices are linearly regressed.

(4) U=TB

(5) $$B={\left(T^{T}T\right)}^{-1}T^{T}Y$$

In the prediction, the *T*_unknown_ of the unknown sample spectral matrix *X*_unknown_ is obtained from *P*, and then, the predicted value matrix is obtained from Equation (6).

(6) $$Y_{\mathrm{unknown}}\;=\;T_{\mathrm{unknown}}\mathrm{BQ}$$

For a PLS model, according to Equations (7) and (8), the decrease of the value of the root mean square error of cross-validation (RMSECV) and the root mean square error of prediction (RMSEP) indicates an increase of the model prediction accuracy.

(7) $$\mathrm{RMSECV}=\sqrt{\frac{{\sum_{i=1}^{n}\left(y_{i,\mathrm{actual}}-y_{i,\mathrm{predicted}}\right)}^{2}}{n-1}}$$

(8) $$\mathrm{RMSEP}=\sqrt{\frac{{\sum_{i=1}^{n}\left(y_{i,\mathrm{actual}}-y_{i,\mathrm{predicted}}\right)}^{2}}{m-1}}$$

Therefore, as a good PLS model, values of RMSECV and RMSEP are expected to be as small as possible. In addition, according to Equation (9), the closer the coefficient of determination (*R*^2^) is to 1, the more accurate the PLS model is.

(9) $$R^{2}=1-\frac{\sum_{i=1}^{n}{\left(y_{i,\mathrm{actual}}-y_{i,\mathrm{predicted}}\right)}^{2}}{{\sum_{i=1}^{n}\left(y_{i,\mathrm{actual}}-{\bar{y}}_{i,\mathrm{actual}}\right)}^{2}}$$

**Figure 8:**
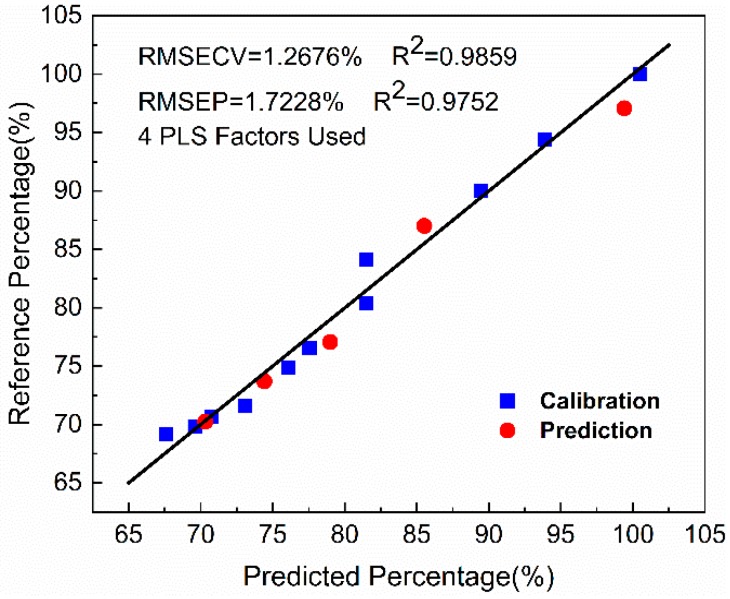
Reference versus predicted degree of PP degradation based on the partial least squares (PLS) model of the calibration and prediction set. RMSECV = the standard error of cross-validation; RMSEP = the standard error of prediction.

Reference versus predicted degree of PP degradation based on the PLS model is shown in Figure 8. The *R*^2^ of the calibration is 0.9859, and RMSECV is 1.2676%. In addition, the *R*^2^ of the prediction is 0.9752, and RMSEP is 1.7228%. These results demonstrate the accuracy of the model prediction. Furthermore, this model prediction ability can achieve the measurement requirements in practical engineering applications.

## 4. Conclusions

Raman spectroscopy coupled with chemometrics methods was used to effectively monitor the degradation of PP under multiple extrusions in real time. The decreases of in-line Raman intensities of the –CH_2_– group and the C-C indicated the scission of PP chains. The decrease of in-line Raman intensities of the –CH_3_ group demonstrated that free radicals terminated with oxygen-containing functional groups instead of –CH_3_ groups. The decreased trend of the in-line Raman intensity of the -CH_2_- group showed that long chains were more easily damaged in the extrusion. These results indicated that an in-line Raman spectroscopy measurement can monitor the degradation in the extrusion more quickly, cost-effectively and without damage. Besides, in combination with PLS, the degree of PP degradation under multiple extrusions measured by the GPC was predicted by the in-line Raman spectroscopy in real time. For the PLS model, *R*^2^ of the calibration and RMSECV were 0.9859 and 1.2676%, *R*^2^ of the prediction and RMSEP were 0.9752 and 1.7228%, which demonstrated the accuracy of the model. It proved that the in-line Raman spectroscopy measurement can monitor the degradation, and what is more, it is capable to quantify the degree of degradation during the extrusion in real time. Thus, in the extrusion process, the in-line Raman spectroscopy measurement was proved to be an accurate and highly effective tool for monitoring the quality of products in real time, which can avoid the production of unqualified products.

## Figures and Tables

**Table 1:** Vibrational modes for Raman Bands of PP [28].

Raman Characteristic Band (cm^−1^)	Vibration Modes
832	C–C stretching, CH_3_ rocking
967	C–C stretching, CH_3_ rocking
1150	C–C stretching, CH bending
1324	CH stretching, CH_2_ wagging, CH_3_ bending
1454	CH_2_ bending, CH_3_ asymmetric bending

**Table 2:** The linear correlation coefficient between the Raman intensity of the PP main characteristic band and the degree of degradation.

**Raman main characteristic band of PP/cm^−1^**	832	967	1150	1324	1454
**The linear correlation coefficient (R)**	0.936	0.912	0.955	0.926	0.958
